# Comprehensive evaluation of methods to assess overall and cell-specific immune infiltrates in breast cancer

**DOI:** 10.1186/s13058-019-1239-4

**Published:** 2019-12-26

**Authors:** Iris Nederlof, Davide De Bortoli, Yacine Bareche, Bastien Nguyen, Michiel de Maaker, Gerrit K. J. Hooijer, Laurence Buisseret, Marleen Kok, Marcel Smid, Gert G. G. M. Van den Eynden, Arie B. Brinkman, Jan Hudecek, Jan Koster, Christos Sotiriou, Denis Larsimont, John W. M. Martens, Marc J. van de Vijver, Hugo M. Horlings, Roberto Salgado, Elia Biganzoli, Christine Desmedt

**Affiliations:** 1 Department of Pathology, Amsterdam University Medical Centre, Meibergdreef 9, 1105 AZ Amsterdam, The Netherlands; 2 Division of Molecular Pathology, The Netherlands Cancer Institute, Plesmanlaan 121, 1066 CX Amsterdam, The Netherlands; 3 Unit of Medical Statistics, Biometry and Bioinformatics “Giulio A. Maccacaro,” Department of Clinical Sciences and Community Health and DSRC, University of Milan, Campus Cascina Rosa, Fondazione IRCCS Istituto Nazionale Tumori, Milan, Italy; 4 J.C. Heuson Breast Cancer Translational Research Laboratory, Université Libre de Bruxelles, Institut Jules Bordet, 1000 Brussels, Belgium; 5 Departments of Medical Oncology and Tumor Biology and Immunology, The Netherlands Cancer Institute, Amsterdam, The Netherlands; 6 Department of Medical Oncology and Cancer Genomics Centre Netherlands, Erasmus MC Cancer Institute, Erasmus University Medical Center, 3015 CN Rotterdam, The Netherlands; 7 Department of Pathology, GZA-ZNA Ziekenhuizen, Wilrijk, Belgium; 8 Department of Molecular Biology, Nijmegen Centre for Molecular Life Sciences, Faculty of Science, Radboud University, 6500 HB Nijmegen, The Netherlands; 9 Department of Research IT, The Netherlands Cancer Institute – Antoni van Leeuwenhoek, Amsterdam, The Netherlands; 10 Department of Oncogenomics, Amsterdam University Medical Centre, Meibergdreef 9, 1105 AZ Amsterdam, The Netherlands; 11 Pathology Department, Institut Jules Bordet, 1000 Brussels, Belgium; 12 Division of Research, Peter MacCallum Cancer Centre, University of Melbourne, Melbourne, Victoria Australia; 13 Department of Oncology, Laboratory for Translational Breast Cancer Research, KU Leuven, Leuven, Belgium

**Keywords:** Immune infiltrate, Breast cancer, Benchmarking, Methodology, Tumor infiltrating lymphocytes, Transcriptome, Methylome, Microscopy, Digital pathology

## Abstract

**Background:**

Breast cancer (BC) immune infiltrates play a critical role in tumor progression and response to treatment. Besides stromal tumor infiltrating lymphocytes (sTILs) which have recently reached level 1B evidence as a prognostic marker in triple negative BC, a plethora of methods to assess immune infiltration exists, and it is unclear how these compare to each other and if they can be used interchangeably.

**Methods:**

Two experienced pathologists scored sTIL, intra-tumoral TIL (itTIL), and 6 immune cell types (CD3^+^, CD4^+^, CD8^+^, CD20^+^, CD68^+^, FOXP3^+^) in the International Cancer Genomics Consortium breast cancer cohort using hematoxylin and eosin-stained (*n* = 243) and immunohistochemistry-stained tissue microarrays (*n* = 254) and whole slides (*n* = 82). The same traits were evaluated using transcriptomic- and methylomic-based deconvolution methods or signatures.

**Results:**

The concordance correlation coefficient (CCC) between pathologists for sTIL was very good (0.84) and for cell-specific immune infiltrates slightly lower (0.63–0.66). Comparison between tissue microarray and whole slide pathology scores revealed systematically higher values in whole slides (ratio 2.60–5.98). The Spearman correlations between microscopic sTIL and transcriptomic- or methylomic-based assessment of immune infiltrates were highly variable (*r* = 0.01–0.56). Similar observations were made for cell type-specific quantifications (*r* = 0.001–0.54). We observed a strong inter-method variability between the omics-derived estimations, which is further cell type dependent. Finally, we demonstrated that most methods more accurately identify highly infiltrated (sTIL ≥ 60%; area under the curve, AUC, 0.64–0.99) as compared to lowly infiltrated tumors (sTIL ≤ 10%; AUC 0.52–0.82).

**Conclusions:**

There is a lower inter-pathologist concordance for cell-specific quantification as compared to overall infiltration quantification. Microscopic assessments are underestimated when considering small cores (tissue microarray) instead of whole slides. Results further highlight considerable differences between the microscopic-, transcriptomic-, and methylomic-based methods in the assessment of overall and cell-specific immune infiltration in BC. We therefore call for extreme caution when assessing immune infiltrates using current methods and emphasize the need for standardized immune characterization beyond TIL.

## Background

In breast cancer (BC), the presence of immune infiltrate and its composition affects prognosis and treatment efficacy, including response to novel immunotherapies [1–5]. Specifically, increased levels of stromal tumor infiltrating lymphocytes (sTILs) are associated with response to neoadjuvant chemotherapy and prognosis in triple negative BC (TNBC) patients [4, 6–11]. In this context, sTIL has now been recognized as a valid prognostic biomarker by the expert panel of the 16th St. Gallen Breast Cancer Conference. Clinical trials investigating immunotherapies in BC are also using TIL or CD8^+^ T cell scores either for screening patients (e.g., NCT02997995) or as an endpoint (e.g., NCT03875573, NCT03815890, NCT03395899). Reliable methods to estimate the amount and composition of the immune infiltrate are therefore critical, for cross-study comparisons and future biomarker development.

Over the past years, several waves of technology have advanced the quantification and characterization of the immune infiltrate in solid tumors. Pathologists have developed methods to study the immune composition through the microscope [12, 13], while advances in computational biology have enabled the inference of cell type composition of solid tumors by utilizing bulk transcriptomic and methylomic data [14]. Currently, these methods are often used interchangeably in translational and fundamental research, assuming that they are providing similar information. However, the different methods do have different properties and it may be challenging to directly compare methods because of this. To the best of our knowledge, a detailed comprehensive comparison of these methods, including cell type inference using pathology, is still missing in BC, partly due to the lack of centralized microscopic, transcriptomic, and methylomic data.

The primary objective of the current study was therefore to compare the estimations of overall and cell-specific immune infiltration obtained by microscopic, transcriptomic, and methylomic methods in the International Cancer Genomics Consortium (ICGC) BC cohort [15–17]. The secondary objective was to evaluate the reliability of the different methods to classify tumors as highly or poorly infiltrated [10], as stratification of patients according to severity of sTIL infiltration has proven prognostic importance and may become a clinical biomarker in the near future [4, 10, 18].

## Methods

### Patients and dataset

This study is established on the ICGC BC cohort (https://dcc.icgc.org/) including 548 primary samples of female patients, for whom transcriptomic and methylomic data were available for 257 and 318 patients, respectively [15]. Data access was granted by ICGC. The generated pathology data, the type of data available for each patient, and the distribution of clinical and pathological characteristics are available at https://doi.org/10.6084/m9.figshare.8234246.

### Quantification of the tumor immune infiltrate

We collected tissue microarrays (TMAs; 3 cores/tumor, *n* = 254), whole slides (WS) for IHC (*n* = 82), and hematoxylin and eosin-stained WS (H&E; *n* = 243). TMAs and WS were stained for CD3, CD4, CD8, CD20, CD68, and FOXP3. Specific antibody clones, dilutions, and incubation times are listed in Additional file 1: Table S1. H&E slides were used to assess stromal TIL (sTIL) and intra-tumoral TIL (itTIL) for the whole slides and the TMAs. Two experienced pathologists (RS, HMH) scored all slides using the online pathology platform Slidescore [19] and reported the percentage of positive immune cells in the stromal and intra-tumoral compartment for each H&E- or IHC-stained slide, according to the existing guidelines [13]. A two-step digital image analysis (DIA) was performed using the Visiopharm Integrator System Software (VIS; Visiopharm A/S, Hoersholm, Denmark) using two optimized applications within the software to recognize positive DAB staining and tissue versus non-tissue. First, an application was used to detect the tissue and remove artifacts. With the second application, the positive-stained area (IHC-stained) was detected and the output variable is the positive area. Detection of, for example, CD3 positivity is based on the HDAB-DAB color deconvolution band. For all samples, the same threshold of positivity was kept.

For the computational analysis, we included methods that provided an estimation of the immune infiltration with respect to the entire tumor (Table 1, Additional file 1: Table S2). This list includes techniques used regularly for cell type inference. It should not been seen as exhaustive as new techniques are constantly being developed. We have included methods based on gene expression and methylation profiles, as both allow to study sample composition. Methods that estimate cellular populations from bulk data can be bluntly divided into two categories:

1. Those based on marker genes, providing an independent (semi-quantitative) assessment for each cell type, enabling comparison between samples but not within samples
2. Those based on deconvolution algorithms, inferring cell type fractions, enabling comparison between and within samples [14, 30]

**Table 1:** Computational and microscopic methods used for estimation of overall infiltration and calculation of specific cell populations

Method	Approach	Description	Overall immune score	Cell populations
H&E whole slide (WS)	Microscopy based	Pathology TIL scores	TIL scores	NA
IHC whole slide (WS)	Microscopy based	Pathology scores with IHC	Summed lymphocyte fractions	Yes
Whole slide digital (digWS)	Microscopy based	Visiopharm digital scores IHC	NA	Yes
H&E tissue microarray (TMA)	Microscopy based	Pathology TIL scores	TIL scores	NA
IHC tissue microarray (TMA)	Microscopy based	Pathology scores IHC	Summed lymphocyte fractions	Yes
TMA digital (digTMA)	Microscopy based	Visiopharm digital scores IHC	Summed lymphocyte fractions	Yes
Absolute CIBERSORT (aCBS) [20]	Deconvolution	Cell fractions, absolute mode used	Summed lymphocyte fractions	Yes
quanTIseq, lsfit (qSEQ) [21]	Deconvolution	Cell fractions, absolute	Summed lymphocyte fractions	Yes
MCP-counter (MCP) [22]	Gene marker	Arbitrary units	NA	Yes
xCell [23]	Gene marker	Arbitrary units	NA	Yes
EPIC [24]	Deconvolution	Cell fractions, absolute	Summed lymphocyte fractions	Yes
MethylCIBERSORT (metCBS) [25]	Deconvolution	Cell fractions, absolute	Summed lymphocyte fractions	Yes
TIL rna score (TILrna) [26]	Gene marker	TIL associated gene signature	TIL signature	NA
meTIL [27]	Gene marker	TIL methylation profile	TIL signature	NA
Cell signatures Davoli et al. [28]	Gene marker	Computed gene signature	NA	Yes
Cell signatures Danaher et al. [29]	Gene marker	Computed gene signature	NA	Yes

To provide potential validation for immune infiltration, we calculated gene signatures specific for immune cell activity, namely cytolytic activity (CytAct) and interferon-gamma (IFNg) [31, 32]. All fractions of TIL and cell populations for each method are available at https://doi.org/10.6084/m9.figshare.8234246.

Details for each method and processing are available in Additional file 1, which also includes Additional file 1: Tables S2 and S3.

### Statistical analysis

Statistical analyses were performed using R version 3.5.1. Values for immune infiltration were log transformed with an offset of 0.05. The agreement between observers and methods was assessed using the Bland-Altman method and Passing-Bablok regression analyses. Specifically, the geometric mean of the two scores from the same stained tumor section (*x* axis) is plotted against the ratio between the two methods or observers (*y* axis), considering the overall geometric mean of the ratios (center line) and the approximate 95% limits of agreement (horizontal lines), and Loess fitted curves were incorporated. Analysis was performed on all samples with available information for the methods taken into consideration. The concordance correlation coefficient (CCC) was used as a summary measure of reproducibility between observations [33] for each cell type. To establish the contribution of the itTIL and sTIL values to all TIL, a combined “averaged” TIL score was calculated by taking the arithmetic mean of the sTIL and itTIL scores for each sample and the Passing-Bablok regression was used for the comparison between TIL scores.

To assess replicability, correlations between methods for tumor immune infiltration were measured using the non-parametric Spearman’s rho coefficient, and a Loess smoothing was used for flexible interpolation. The 95% confidence intervals (CI) were calculated using the bootstrapping procedure with 1000 bootstrap samples (overlapping the results from the asymptotic approximation).

Both the Spearman’s rho coefficient and Linn’s CCC were interpreted according to qualifiers as “very poor” (< 0.20), “poor” (0.20–0.40), “moderate” (0.40–0.60), “good” (0.60–0.80), and “very good” (0.80–1.00) [34].

To distinguish the highly and poorly infiltrated tumors, the (calculated) overall immune infiltration value (available at https://doi.org/10.6084/m9.figshare.8234246) from each method was utilized. The thresholds used for the categorization are based on the published meta-analysis on BC infiltration [10]: sTIL ≤ 10% for poorly infiltrated tumors, 11–59% for intermediately infiltrated tumors, and ≥ 60% for highly infiltrated tumors. To evaluate if different methods would classify the same tumor as poorly or highly infiltrated, receiver operator characteristic curves were generated and area under the curves (AUCs) of each classifier were calculated using pROC package version 1.13.0. All tests were two-sided.

## Results

### Assessment of immune cells on H&E and IHC whole slides

**Fig. 1:**
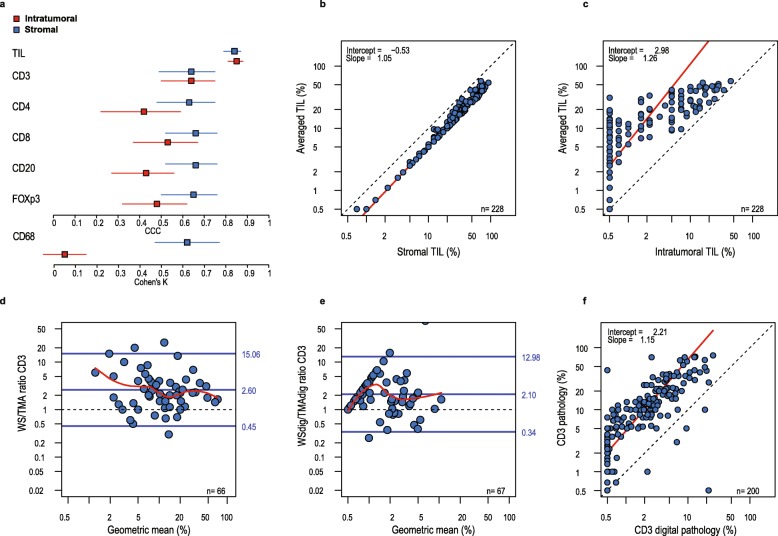
Reliability of standard microscopic pathology and digital analysis to estimate the immune composition. **a** Forest plots representing estimated concordance correlation coefficients with 95% confidence interval (CI) for each pairing between pathologists (inter-observer agreement) on H&E and whole slides (WS). CCC (95% CI): sTIL, 0.84 (0.79–0.87); itTIL, 0.85 (0.81–0.88); sCD3, 0.64 (0.49–0.75); itCD3, 0.64 (0.50–0.75); sCD4, 0.63 (0.48–0.75); itCD4, 0.42 (0.22–0.59); sCD8, 0.66 (0.52–0.76); itCD8, 0.53 (0.37–0.67); sCD20, 0.66 (0.52–0.76); itCD20, 0.43 (0.27–0.56); sFOXP3, 0.65 (0.50–0.76); and itFOXP3, 0.48 (0.32–0.62). Cohen’s *K* for the macrophages staining (CD68) based on 4 infiltration categories (nil, mild, moderate, and severe). **b** The Passing-Bablok regression between the averaged (averaged sTIL and itTIL) TIL and sTIL (*r* = 0.84). **c** The Passing-Bablok regression between the global TIL and itTIL (*r* = 0.37). **d** The Bland-Altman analysis of the CD3 score agreement between TMA and WS. **e** The Bland-Altman analysis for agreement between CD3 whole slide digital pathology and CD3 TMA digital pathology. **f** The Passing-Bablok between CD3 assessment by pathologists and digital pathology

Two experienced pathologists scored itTIL, sTIL, and six immune cell types (CD3^+^, CD4^+^, CD8^+^, CD20^+^, CD68^+^, FOXP3^+^) in an invasive primary BC cohort [15]. In line with previous reports [35–39], we observed a very good inter-observer CCC both for sTIL and itTIL (0.84 and 0.85, respectively, Fig. 1a). The limits of agreement showed a fair relative precision between measurements, and no major constant (intercept) or proportional (slope) drift between the two pathologists (Additional file 1: Figure S1). A very good concordance was observed between the stromal and averaged TIL (CCC 0.84, Fig. 1b), while by contrast, a poor concordance was observed between the intra-tumoral and averaged TIL score (CCC 0.37, Fig. 1c). These results show that immune infiltration in BC is mainly localized in the stromal compartment and is not greatly interfered by intra-tumoral infiltration. Of note, some tumors with a high overall infiltration could still lack itTIL, as depicted in Fig. 1c, and include most probably tumors where the immune infiltrate is restricted to the stroma or to the tumor margin. The inter-observer analysis demonstrated a moderate CCC for the immunohistochemical (IHC) assessments, where stromal scoring performed overall better and more precisely than intra-tumoral scoring (Fig. 1a). All stromal IHC CCC values were lower for immune cell subtypes than for sTIL and itTIL assessment on H&E, ranging from 0.63 to 0.66 (Fig. 1a). The intra-tumoral IHC methods had a CCC below 0.6, except for itCD3 (CCC = 0.64). We therefore only considered the more reliable and abundant stromal estimates for further analyses.

### Comparison of tumor immune infiltration estimates between tissue microarrays and whole slides (H&E and IHC)

**Fig. 2:**
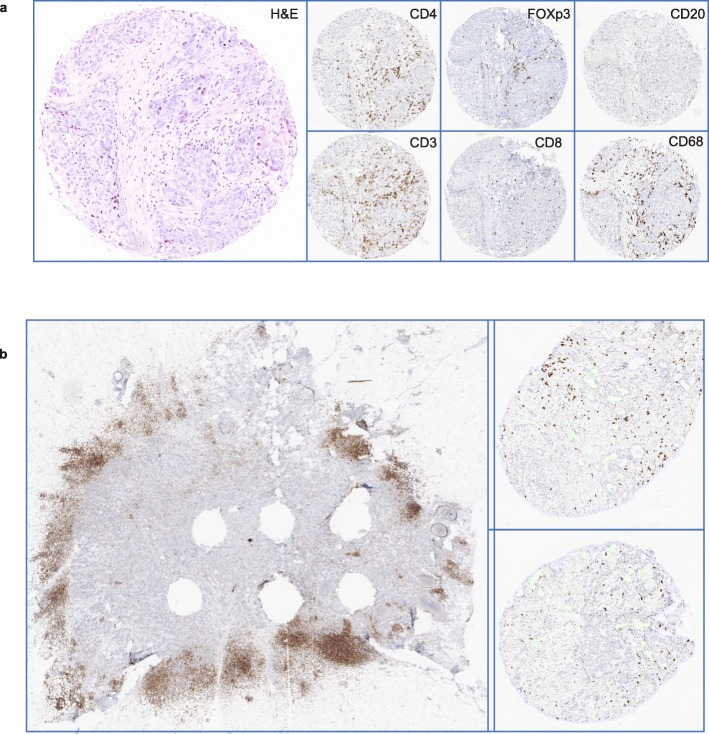
Immunohistochemical assessment of immune cells. **a** Immunohistochemical staining for 6 immune markers and the hematoxylin and eosin-stained corresponding tissue microarray (TMA) core. **b** Whole slide and corresponding TMAs, stained for CD3, for patient PD13760

**Fig. 3:**
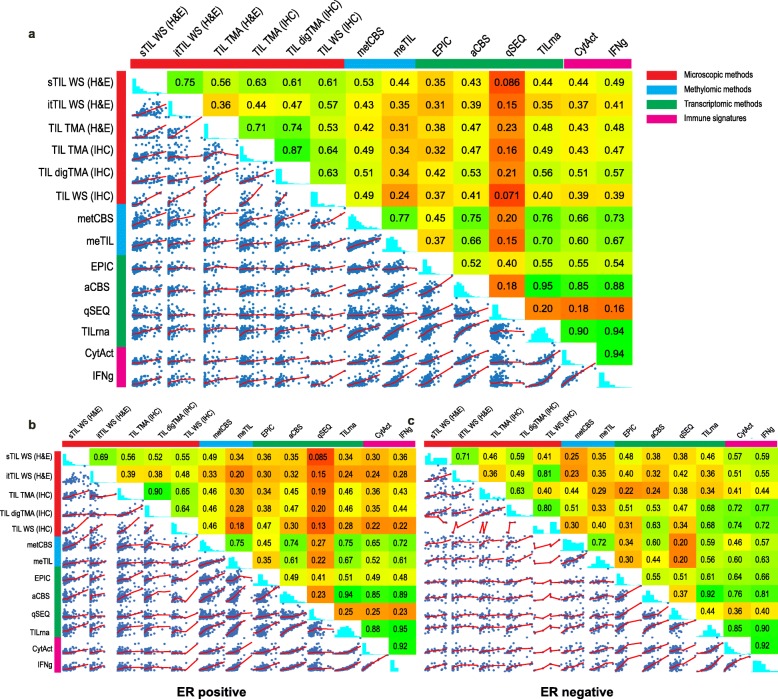
Methods to assess overall infiltration. **a** Matrix plot of Spearman’s correlations for the methods providing information on overall immune infiltration; tumor infiltrating lymphocytes (TILs) or the sum of T cells and B cells was taken to derive a TIL fraction. **b** Matrix plot for Spearman’s correlations of the methods providing information on overall immune infiltration in ER-positive tumors and **c** ER-negative tumors. aCBS, absolute CIBERSORT [20]; digTMA, tissue microarray scored by Visiopharm (digital analysis); MCP, MCP-counter [22]; meTIL, methylation TIL score [27]; metCBS, methylCIBERSORT [25]; itTIL, intra-tumoral TIL on H&E; qSEQ, quanTIseq [21]; sTIL, stromal TIL on H&E; TILrna, TIL score based on transcriptome [26]; TMA (H&E), sTIL scored on TMA; TMA (IHC), tissue microarray scored by pathologists with CD3 and CD20 markers to calculate the sTIL; WS (IHC), whole slide immunohistochemistry of CD3 and CD20 by pathologists

When stromal TIL was scored on both whole H&E slides and TMA H&E, the correlation was moderate (0.56) (Fig. 3). The CCC between WS and TMA was only 0.26, where overall a lower (5.98× lower) percentage was scored on H&E TMAs compared to whole H&E slides (Additional file 1: Figure S2a). We characterized six immune features both on TMA and WS (Fig. 2a). The immune infiltrate scores were systemically higher on WS as compared to TMA, as depicted in Fig. 1d for the most abundant immune cell population, i.e., CD3^+^, and in Additional file 1: Figure S2a for the other immune cells. The CCCs between WS and TMA were globally poor and ranged between 0.21 for CD4^+^ and 0.43 for FOXP3^+^ cells (Additional file 1: Figure S2b). We further evaluated both TMA and WS using digital pathology and confirmed the higher level of immune infiltration estimated with WS as compared to TMA (Fig. 1e, Additional file 1: Figure S2c), highlighting the spatial heterogeneity of the tumor immune microenvironment (Additional file 1: Figure S2b). Figure 2b depicts one sample that shows high infiltration on the WS and limited infiltration in the TMA. In line with previous reports [35], we observed a moderate correlation between human and digital assessment of CD3^+^ (CCC = 0.42, Fig. 1f), yet the digital evaluation showed a lower estimation of all immune cells compared to pathologists (Additional file 1: Figure S2d,), suggesting a relative human overestimation or digital underestimation of tumor immune infiltration.

### Comparison of microscopic, transcriptomic, and methylomic evaluation of overall tumor immune infiltration

To evaluate if different data types could estimate overall immune infiltration consistently, we compared the (calculated) score of several microscopic-, transcriptomic-, and methylomic-based methods. In addition, two inflammatory gene signatures (interferon-gamma and cytolytic activity) were calculated (pink category in Fig. 3), as these often correlate to tumor immune infiltration and can provide information on the status of the tumor microenvironment [31, 32]. A description of all methods is provided in Table 1 and Additional file 1: Table S2. First, while the Spearman correlations between microscopic (red) and all methylomic (blue) or transcriptomic (green) estimates were poor to moderate, our analysis showed that stromal infiltration correlates better with all other methods, including transcriptomic and methylomic methods, as compared to the intra-tumoral infiltration (Fig. 3a). We tested the possibility that higher itTIL may lead to more pronounced inflammatory gene expression than sTIL as itTIL may have a crucial anti-tumor role [40–44]. Yet, we observed no higher correlation between intra-tumoral infiltration and inflammation-associated signatures (immune signatures, pink label Fig. 3a). Secondly, a good correlation was observed between stromal microscopy assessment on H&E-stained (sTIL) and IHC-stained WS, where the sum of T (CD3^+^) and B (CD20^+^) cells were considered (*r* = 0.61). Thirdly, the correlation with the immune gene signatures, cytolytic activity (*r* = 0.51) and IFNg (*r* = 0.57), improved slightly when the infiltrate was scored with digital pathology compared to the other microscopic (red) methods (Fig. 3a). This is most probably because both the immune signatures (CytAct and IFNg) and the digital assessment of the TMA do not consider the type of infiltrates (intra-tumoral vs stromal). The same trend was observed between the digital assessment of the TIL on TMA (digTMA) and transcriptomic TIL methods (green), where the Spearman correlations with EPIC (*r* = 0.42), aCBS (*r* = 0.53), and TILrna (*r* = 0.56) were again the highest compared to all other microscopic (red) methods. Fourthly, as expected, methods using the same modality for input of data showed better correlations. For example, several transcriptomic estimates showed a very good correlation with each other (*r* > 0.80), and methylCIBERSORT [25] and the methylomic TIL score, meTIL [27], showed a reassuring good agreement (*r* = 0.77). The correlation between transcriptomic and methylomics was variable, but methylCIBERSORT showed good correlations with absolute CIBERSORT (*r* = 0.75) and TILrna [26] (*r* = 0.76), and similar observations could be made for meTIL and CIBERSORT(*r* = 0.66) and meTIL and TILrna (*r* = 0.70). Fifthly, of the transcriptomic and methylomic methods, TILrna, methylCIBERSORT, and absolute CIBERSORT [20] showed the highest correlations with microscopy scores, though the correlations were still not perfect with the highest being 0.56 between digTMA and TILrna, 0.53 considering sTIL and methylCIBERSORT, and 0.53 between digTMA and absolute CIBERSORT. These numbers were however in line with a recently published lung cancer study [45]. Finally, of the methods that predict global immune infiltration based on the transcriptome (green label in Fig. 3a), TILrna showed the highest correlations with the various immune gene signatures (pink label, *r* = 0.90–0.94), while quanTIseq [21] showed the poorest correlations (*r* = 0.16–0.18). Similar analyses were further carried on separately for ER-negative and ER-positive tumors as the biological significance of the immune infiltrate may be different [46, 47] (Fig. 3b, c). The correlations for the microscopic- versus methylomic- and transcriptomic-based methods were in general slightly higher in the ER-negative compared to the ER-positive subgroup. Nevertheless, compared to all samples, the ER-negative tumors did not necessarily show higher correlations.

**Fig. 4:**
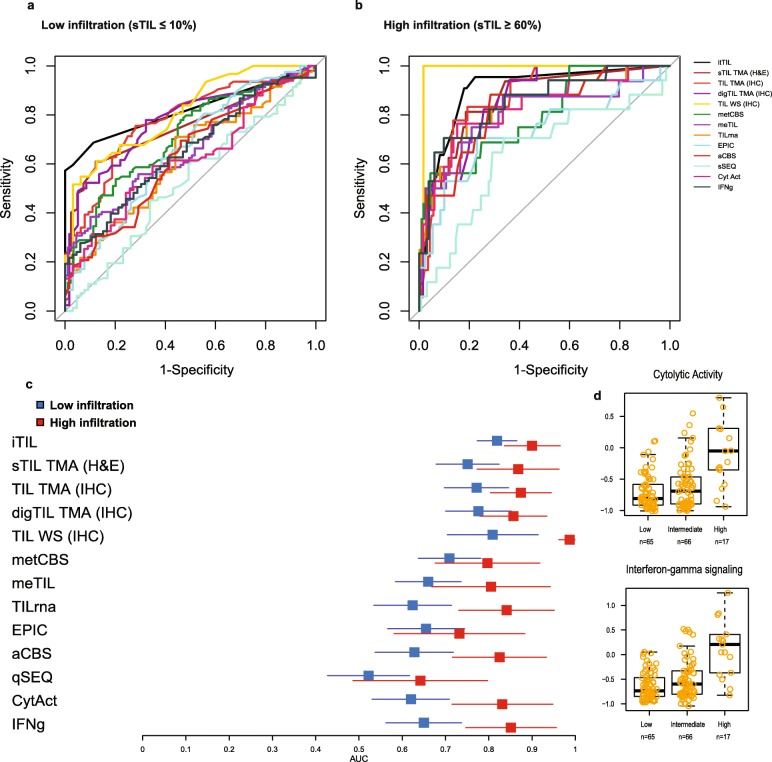
Identifying lowly and highly infiltrated tumors. The ROC curves for the different TIL methods **a** to classify lowly infiltrated tumors compared to the rest (blue color) and **b** to classify highly infiltrated tumors compared to rest (red color). The corresponding AUCs from **a** (blue color) and **b** (red color) are depicted in **c**. The distribution of TIL scores for the TIL methods and inflammatory signatures—cytolytic activity and interferon-gamma—is depicted in **d** for the low (≤ 10%), medium (11–59%), and high (≥ 60%) infiltrated tumors according to stromal TIL scores

Following the analysis at the continuous level (Fig. 3a–c), we further aimed to investigate the ability of the methods to identify lowly infiltrated (sTIL ≤ 10%) and highly infiltrated (sTIL ≥ 60%) tumors as defined by Denkert and colleagues [10]. TIL is currently not used as a classifier in the clinic, yet the stratification of patients based on TIL has provided important prognostic information in clinical studies [4, 18]. To this end, a ROC curve analysis was performed (Fig. 4a, b) and most methods performed moderate to very poor in recognizing the lowly infiltrated tumors (Fig. 4a, c blue boxes). The itTIL score showed the highest area AUC for the lowly infiltrated tumors (Fig. 4c, blue boxes), and the methylomic-based TIL scores had a slightly higher AUC as compared to the transcriptomic-based methods, although their confidence intervals were overlapping. ROC analyses further showed that most methods can more accurately identify highly infiltrated tumors as compared to lowly infiltrated tumors (Fig. 4b, c red boxes). Here, the highest AUCs were still based on the microscopy methods, but methylomic- and transcriptomic-based methods also showed fair to high AUCs. Of interest, highly infiltrated tumors have the highest expression of inflammatory signatures, like interferon-gamma and cytolytic activity (Fig. 4d), yet lowly infiltrated tumors could also show a wide range of these inflammatory gene expressions.

### Comparison of microscopic, transcriptomic, and methylomic evaluation of cell-specific immune infiltration

**Fig. 5:**
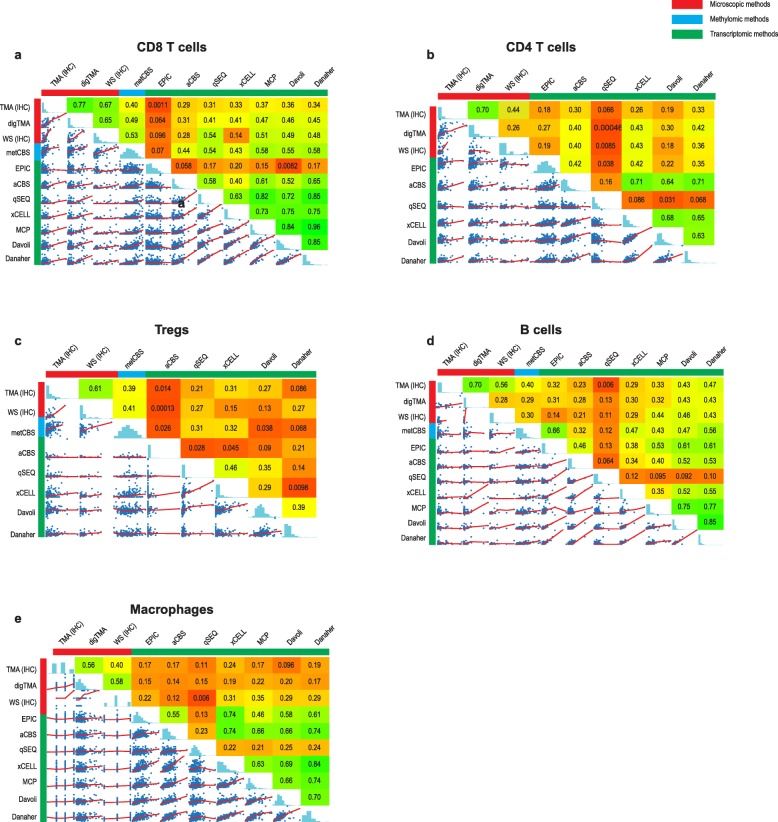
Methods to assess cell-specific infiltration. Spearman’s correlation plots for CD8 T cells (**a**), CD4 T cells (**b**), regulatory T cells (Tregs) (**c**), B cells (**d**), and macrophages (**e**). aCBS, absolute CIBERSORT [20]; digTMA, tissue microarray scored by Visiopharm (digital analysis); MCP, MCP-counter [22]; meTIL, methyl TIL score [27]; metCBS, methylCIBERSORT [25]; itTIL, intra-tumoral TIL on H&E; qSEQ, quanTIseq [21]; sTIL, stromal TIL on H&E; TILrna, TIL score based on transcriptome [26]; TMA IHC, tissue microarray scored by pathologists; WS IHC, whole slide immunohistochemistry by pathologists

Several observations could be made from the comparison between microscopic and computational methods that quantify specific immune cell types with transcriptomic or methylomic data (Table 1, Fig. 5a–e). Firstly, the correlations between the microscopic evaluations and the transcriptomic and methylomic data were always < 0.60, with CD8^+^ T cells showing the highest (Fig. 5a) and macrophages (Fig. 5e) the lowest correlation between microscopic and methylomic or transcriptomic methods, respectively. Of interest, there was no systematic increase in the correlation coefficients when considering WS instead of TMA scores. Of note, when we examined the CCC between methods for specific cell types, poor concordance (< 0.3) was found for all methods (data not shown). Secondly, most omics-derived methods showed large inconsistencies between cell types regarding their correlation with microscopy. For example, while quanTIseq showed very poor correlation with sTIL (0.09; Fig. 3a) and CD4^+^ cells (0.01, WS evaluation; Fig. 5b), the correlations for CD8^+^ were moderate (0.54, WS evaluation, Fig. 5a). Altogether, these analyses highlight the variable correlation between the different omics-derived methods and microscopic assessments, which further vary according to the immune cell type.

## Discussion

In this study, we have comprehensively demonstrated that methods of the same modality (microscopy, transcriptomic, or methylomic based) to estimate overall infiltration show reassuring correlations, but the correlations deteriorate when comparing between modalities. In addition, we quantified specific immune cell types and observed a strong heterogeneity in the correlations between the microscopic- and omics-based estimates, and also between the different omics-based estimates. For each cell type, the best method to use may be different. Our analysis clearly shows that different transcriptomic and methylomic methods have limitations in estimating immune infiltration, as correlations with microscopy-based methods do not exceed 0.6. As the pathology assessment of TIL has reached level 1B evidence with new studies confirming its value for the patient [4], the fact that correlations are not high may warrant extra caution when using non-pathology methods as these may measure different characteristics of the immune infiltrate. These findings might be explained by the fact that transcriptomic and methylomic data are not perfectly representing protein expression of immune cells. In addition, regular bulk transcriptome or methylome analysis does not detect heterogeneity within a sample and ignores the localization of cells [48]. An example as depicted in Fig. 2b emphasizes the importance of spatial heterogeneity, but also raises the question about the functionality of the observed immune infiltrate. Importantly, transcriptomic or methylomic methods may be biased towards a specific cell state, as they are often based on cells challenged with experimental processes (e.g., tissue digestion or flow cytometry) or from different origin (e.g., peripheral blood or different tumors), which may contribute to the observed discordance with microscopy.

More research into the contribution and spatial distribution of specific immune cells in the context of clinical outcome is warranted to understand beneficial and detrimental immune cell profiles. Spatially resolved methods, measuring genetic and phenotypic diversity, would support advances for clinical studies [49]. Moreover, integration of deep learning approaches with morphological features, in conjunction with genomic-derived data, will probably be needed to derive a full comprehensive evaluation of the immune environment in solid tumors. Our data provided us with insightful observations that can guide future research using digital image analysis. First, we showed that stromal evaluation of several immune cell types is reliable with an acceptable concordance observed between pathologists, especially for stromal infiltration. Second, values may also be estimated with a digital approach, taking into account the systematically lower numerical values for digital estimates [35]. Third, our results raise caution for the evaluation of immune markers on TMA (or biopsies) as small punches of the tumor may obscure information of infiltration and WS showed overall a higher infiltration than TMA. Our results on immune infiltrate are in contrast with several studies which demonstrated that TMAs are reliable for the evaluation of several prognostic epithelial-based tumor markers [50–53].

Our second objective was to stratify patients into lowly infiltrated and highly infiltrated tumors, as more clinical studies showed that sTIL-based stratification could serve as an important prognosticator [4, 18]. We observed that microscopic methods (itTIL, WS, TMA IHC, digTMA) were better in the stratification of patients into lowly or highly infiltrates, as expected due to their common modality as sTIL (as opposed to, for example, gene expression-based methods). We also showed here that the majority of the methods are better in recognizing highly infiltrated tumors as compared to lowly infiltrated tumors. The lower accuracy for identification of lowly infiltrated tumors may be problematic as the majority of the breast tumors will have infiltration above 0% but far below 60%, as shown by Loi et al. [4] in early TNBC. These findings should be taken into consideration in developing inclusion and stratification criteria, as well as endpoints in the context of clinical trials.

Strengths of this study include the large number of patients and extensive central evaluation of immune cells, together with the availability of transcriptomic and methylomic data. A limitation of our study is that tissue analysis (FFPE) and DNA/RNA isolation (FF) were not performed on the exact same area from the tumor. Infiltration may be affected by heterogeneity and partially explain the correlations not exceeding 0.6. We minimized this effect by studying multiple cores spread throughout the tumor in the TMA and large sections of the tumors in WS, far exceeding the area usually evaluated with biopsies. Nevertheless, correlation with various omics-based methods did not systematically increase when considering WS versus TMA. In addition, the digital analyses of the immune cells were calculated as an area, while pathologists report a cellular percentage, leading to pre-analytical factors that affect the results. We were however not able to calculate the cellular percentage due to visual cell segmentation problems. Another limitation is that the omics-based methods considered in this manuscript do not consider the localization of the cells in the tumors. In this context, a recent study conducted in TNBC suggested that transcriptomics might have the potential to derive this spatial information [54]. Finally, this cohort does not provide follow-up or therapy response data. Future studies may test superiority of specific measures with the ultimate goal to better guide precision medicine.

## Conclusion

This study highlights an important heterogeneity in the various estimates of immune infiltrates in BC and calls for caution when used in the clinical context. This study further provides an important resource of multi-level data of the tumor immune microenvironment to researchers for future investigations. Ultimately, there is an urgent need for the development of international guidelines to categorize breast tumors according to their immune infiltrate in both a quantitative and a qualitative manner. Combining the valuable information from multiple methods, e.g., the spatial information from pathology and transcriptomic information on cellular activity, may elucidate the role of immune infiltration in disease progression in a more accurate manner.

## Supplementary information

**Additional file 1: Supplementary Methods. Figure S1.** The inter-observer analysis for all the stromal and intratumoral immune cell scores. **Figure S2.** Comparison between (dig) WS and (dig)TMA. **Table S1.** The procedures, clones, manufacturer and dilution used for the immunohistochemistry on tissue micro array (TMA) and Whole Slides (WS). **Table S2.** Description of all methods used in the manuscript to estimate immune cell infiltration. **Table S3.** Overview of used cell fractions for overall immune infiltration and specific immune subtypes.

## Data Availability

The clinical, pathologic, transcriptomic, genomic, and methylation data used in this study was already publicly available upon request to the ICGC data access committee (https://icgc.org/daco). The microscopic data that were generated specifically for this study are included in this published article and its additional information files.

## Abbreviations

AUC: Area under the curve
BC: Breast cancer
CCC: Concordance correlation coefficient
ER: Estrogen receptor
FF: Fresh frozen
FFPE: Formalin-fixed paraffin-embedded
H&E: Hematoxylin and eosin
HER2: Human epidermal growth factor receptor 2
ICGC: International Cancer Genomics Consortium
IHC: Immunohistochemistry
itTIL: Intra-tumoral tumor infiltrating lymphocyte
sTIL: Stromal tumor infiltrating lymphocyte
TIL: Tumor infiltrating lymphocyte
TMA: Tissue microarray
TNBC: Triple negative breast cancer
Tregs: Regulatory T cells
WS: Whole slide
