# Supplementary material for: Comprehensive evaluation of methods to assess overall and cell-specific immune infiltrates in breast cancer
Source: Breast Cancer Res. 2019 Dec 26;21:151. doi: 10.1186/s13058-019-1239-4 (PMC6933637; doi:10.1186/s13058-019-1239-4)
Supplement: Supplementary file 1 — Additional file 1: Supplementary Methods. Figure S1. The inter-observer analysis for all the stromal and intratumoral immune cell scores. Figure S2. Comparison between (dig) WS and (dig)TMA. Table S1. The procedures, clones, manufacturer and dilution used for the immunohistochemistry on tissue micro array (TMA) and Whole Slides (WS). Table S2. Description of all methods used in the manuscript to estimate immune cell infiltration. Table S3. Overview of used cell fractions for overall immune infiltration and specific immune subtypes. [file 13058_2019_1239_MOESM1_ESM.docx]

**ADDITIONAL FILE 1 FOR**

# “Comprehensive evaluation of methods to assess overall and cell-specific immune infiltrates in breast cancer” by Nederlof et al.

A. Supplementary Methods (p1)

B. References (p6)

C. Supplementary Figures (p9)

D. Supplementary Tables (p12)

**A. Supplementary Methods**

**Patients and dataset**

The cohort used in the study is the BASIS breast cancer cohort of the International Cancer Genome Consortium (ICGC) including 560 tumor samples of breast cancer patients ^1^, of whom DNA and RNA were analyzed using whole genome sequencing (WGS), DNA methylation profiling and RNA sequencing. This included 191 ER negative and 357 ER positive tumors, 73 HER2 positive and 457 HER2 negative tumors. 47 tumors were of grade 1, 147 of grade 2 and 152 tumors of grade 3. The BC dataset is hosted by the ICGC and deposited at <https://dcc.icgc.org/>. Detailed clinical and molecular characterization of these participants were previously published ^1^. From the original dataset, the patients with local relapse (n=7), metastasis (n=1), and male gender (n=4) were excluded, leading to a total of 548 female patients with primary breast cancer. RNA sequencing data (all ≥5 million counts assigned) was available for 257 patients, DNA methylation data was available for 318 patients and Whole Genome Sequencing was available for all patients. We collected tissue of the primary tumors for histopathological and immunohistochemical evaluation. Types of data available for each and the distribution of clinical and pathological characteristics are available at https://doi.org/[10.6084/m9.figshare.8234246](https://doi.org/10.6084/m9.figshare.8234246).

**Availability of data**

Data access was granted by the International Cancer Genome Consortium (ICGC). Internal review boards of each participating institution approved collection and use of samples of all patients in this study. Ethical guidelines were in accordance with the Declaration of Helsinki. Informed consent was obtained by the relevant participating institution. IRB approval for this specific study was granted 14-5-2018 by the institutional review board of the Netherlands Cancer Institute where the additional experiments were performed. The dataset supporting the conclusions of this article is available in the ICGC repository, [<https://dcc.icgc.org/projects/BRCA-EU>], and can be requested via the ICGC’s Data Access Compliance Office. The newly generated pathology data are published at https://doi.org/[10.6084/m9.figshare.8234246](https://doi.org/10.6084/m9.figshare.8234246).

**Immunohistochemistry**

The TMAs used in this work were constructed using formalin-fixed, and paraffin-embedded (FFPE) primary invasive breast cancer samples. From the selected (donor) blocks three cores of tumor with thickness of 0.6 mm were collected using a TMA instrument (Beecher Instruments, Silver Springs MD, USA) and inserted in a recipient block. Each recipient block was sectioned at 4 μm, and dried overnight at 37ºC. 4um sections of the whole tissue blocks and TMAs were cut and dried overnight in a 37ºC incubator. All of the staining procedures were performed on a Ventana Benchmark XT (WS) or Ventana Benchmark Ultra (TMA) automated staining instrument (Ventana Medical Systems). The Ventana staining procedure includes deparaffinization of the tissue sections and Heat- Induced Epitope Retrieval using cell conditioning (CC1), followed by incubation with antibodies at the appropriate dilution. Specific antibody clones, dilutions, and incubation times are listed in Supplementary Table 1. Sections were scanned at 20× magnification using a Aperio Scanscope Scanner, using the image format [.svs].

**IHC assessment by pathologists**

Assessment was done according to published guidelines ^2^. The immune cell abundance was estimated by the pathologists as the fraction of cells of the total of stromal cells and as the immune cell fraction of the infiltrating cells in the tumor, defined as the stromal or intra-tumoral infiltration, respectively. For the TMAs a consensus score was provided by two pathologists (RS, HH) for stromal (CD3, CD4, CD8, CD20, CD68, FOXp3) and intra-tumoral (CD3, CD8) infiltration. For the whole slides stromal and intra-epithelial immune infiltration of CD3, CD4, CD8, CD20, CD68 and FOXP3 was independently evaluated by two pathologists (HH, RS) to assess interobserver variability. These values were used for the inter-observer study. Hereafter, the pathologists re-evaluated all highly discordant stromal IHC (arbitrarily defined as >10% difference between pathologists) to strive for a better accuracy of immune characterization for further analysis. New consensus scores were provided for the stromal values. TILs were assessed from the available H&E’s by two pathologists. In the analysis, the geometric mean of the two scores was used. All scores were given on a scale from 0-100%, with the exception of CD68, which was scored in 4 categories (0=null, 1=low infiltration, 2= intermediate infiltration, 3= severely infiltrated).

**IHC assessment by digital pathology**

All TMA cores were evaluated for CD3, CD4, CD8, CD20, and CD68 by digital image analysis using Visiopharm Integrator System software (VIS; Visiopharm A/S, Hoersholm, Denmark). TMAs were dearrayed to evaluate each single core separately. The staining was quantified as the percentage positive area off the total tissue area. In brief, we used an image analysis algorithm developed specifically for recognizing specific lymphocytes and macrophages in Visiopharm. Empty space was automatically excluded from the tissue to avoid contributions from regions of no interest. Cores with artifacts (e.g. folds) were excluded post-processing and not included in the comparative analysis. For the whole slides, CD3 was digitally analyzed according to the same procedure as described for the TMA’s.

**Data processing**

Gene expression values were retrieved as FPKM (Fragments Per Kilobase Million) and transformed to TPM (transcripts per million) values, providing expression data for 44558 genes. If multiple probes mapped to a specific gene, the average signal for a gene was calculated and the probe with the highest signal was included. DNA methylation data was processed in R using the preprocessQuantile() normalization from minfi R package ^3^. CIBERSORT was run using 1000 permutations and quantile normalization was disabled.

**Computational methods to estimate overall immune infiltration and immune activation**

To explore the abundance and characterization of the tumor micro-environment, we searched for several free and publicly available methods and the according reference gene expression profiles of immune cells for bulk-mRNA and methylation. For estimation of overall infiltration, as depicted in Fig. 3 a-c, we included methods that provided an estimation of the lymphocytic immune infiltration with respect to the entire tumor (e.g. summing B cell and T cell fractions). The absolute mode of CIBERSORT (aCBS) ^4^, quanTIseq ^5^ and EPIC ^6^ generate estimations relative to the total amount of sequenced cells, including tumor cells, and therefore provided absolute scores that were interpreted as cell fractions, and could be summed. In addition, a TIL specific marker-gene based approach was included ^7,8^, to estimate TIL abundance between samples. Default settings of the methods, if based on immune cells, were used to obtain fractions of the immune cell population and/or the tumor cell populations. Next, we used the DNA methylation data for epigenomic deconvolution. Methylation profiles are amenable to deconvolution due to their linearity and measurement within the complete (0–1) dynamic range. We employed to this end the recently published method methylCIBERSORT^9^ that provides an absolute score for the cell fractions. For the breast cancer methylation signature breast cancer cell lines ^10^ were used and normalized using single sample Noob method as implemented in the R package minfi ^3^. In addition, we used the published methylome TIL (meTIL) signature ^11^, to estimate TIL abundance from a methylation level. To provide context and potential validation for the immune infiltration, we calculated gene signatures specific for inflammation or immune cell activity (referred to as ‘immune signatures’, pink label Fig, 3a),namely cytolytic activity (Cyt Act) ^12^ and interferon-gamma signaling (IFNg) ^13^.

To study the composition of the immune infiltrate, we investigated the contribution of several immune cells to the infiltration including T cells, CD4 T cells, CD8 T cells, regulatory T cells (Tregs), B cells and macrophages. The absolute modus of CIBERSORT(aCBS) ^4^, methylCIBERSORT (metCBS)^9^, quanTIseq (qSEQ)^5^, EPIC^6^, xCell^14^, and the pathology-based TMA and WS scores. Default settings of the methods, if based on immune cells, were used to obtain fractions of the immune cell population and/or the tumor cell populations. We added immune signature scores for the cell-type populations from 2 different methods^15,16^. All fractions of TIL and cell-populations are listed at https://doi.org/[10.6084/m9.figshare.8234246](https://doi.org/10.6084/m9.figshare.8234246). Details for each method and processing are available in the Supplementary Table 2 and 3 and Table 1.

**B. REFERENCES SUPPLEMENTARY METHODS**

1. Nik-Zainal S, Davies H, Staaf J, et al. Landscape of somatic mutations in 560 breast cancer whole-genome sequences. *Nature*. 2016;534(7605):47-54.

2. Salgado R, Denkert C, Demaria S, et al. The evaluation of tumor-infiltrating lymphocytes (TILs) in breast cancer: recommendations by an International TILs Working Group 2014. *Ann Oncol*. 2015;26(2):259-271.

3. Aryee MJ, Jaffe AE, Corrada-Bravo H, et al. Minfi: a flexible and comprehensive Bioconductor package for the analysis of Infinium DNA methylation microarrays. *Bioinformatics*. 2014;30(10):1363-1369.

4. Newman AM, Liu CL, Green MR, et al. Robust enumeration of cell subsets from tissue expression profiles. *Nat Methods*. 2015;12(5):453-457.

5. Finotello F, Mayer C, Plattner C, et al. quanTIseq: quantifying immune contexture of human tumors. *bioRxiv*. November 2017:223180.

6. Racle J, Jonge K de, Baumgaertner P, Speiser DE, Gfeller D. Simultaneous enumeration of cancer and immune cell types from bulk tumor gene expression data. *Elife*. 2017;6.

7. Smid M, Rodríguez-González FG, Sieuwerts AM, et al. Breast cancer genome and transcriptome integration implicates specific mutational signatures with immune cell infiltration. *Nat Commun*. 2016;7:12910.

8. Massink MPG, Kooi IE, Martens JWM, Waisfisz Q, Meijers-Heijboer H. Genomic profiling of CHEK2*1100delC-mutated breast carcinomas. *BMC Cancer*. 2015;15(1):877.

9. Chakravarthy A, Furness A, Joshi K, et al. Pan-cancer deconvolution of tumour composition using DNA methylation. *Nat Commun*. 2018;9(1):3220.

10. Iorio F, Knijnenburg TA, Vis DJ, et al. A Landscape of Pharmacogenomic Interactions in Cancer. *Cell*. 2016;166(3):740-754.

11. Jeschke J, Bizet M, Desmedt C, et al. DNA methylation-based immune response signature improves patient diagnosis in multiple cancers. *J Clin Invest*. 2017;127(8):3090-3102.

12. Rooney MS, Shukla SA, Wu CJ, Getz G, Hacohen N. Molecular and Genetic Properties of Tumors Associated with Local Immune Cytolytic Activity. *Cell*. 2015;160(1-2):48-61.

13. Ayers M, Lunceford J, Nebozhyn M, et al. IFN-γ–related mRNA profile predicts clinical response to PD-1 blockade. *J Clin Invest*. 2017;127(8):2930-2940.

14. Aran D, Hu Z, Butte AJ. xCell: digitally portraying the tissue cellular heterogeneity landscape. *Genome Biol*. 2017;18(1):220.

15. Danaher P, Warren S, Dennis L, et al. Gene expression markers of Tumor Infiltrating Leukocytes. *J Immunother Cancer*. 2017;5(1):18.

16. Davoli T, Uno H, Wooten EC, Elledge SJ. Tumor aneuploidy correlates with markers of immune evasion and with reduced response to immunotherapy. *Science (80- )*. 2017;355(6322):eaaf8399.

17. Chakravarthy A, Furness A, Joshi K, et al. Pan-cancer deconvolution of tumour composition using DNA methylation. *Nat Commun*. 2018;9(1):3220.

18. Becht E, Giraldo NA, Lacroix L, et al. Estimating the population abundance of tissue-infiltrating immune and stromal cell populations using gene expression. *Genome Biol*. 2016;17(1):218.

**C. Supplementary Figures**

**Supplementary Figure 1.**

**The inter-observer analysis for all the stromal and intratumoral immune cell scores.** Bland-Altman plots show the limits of agreement and are used to compare two observers (RS and HH), for the same variable. Passing-Bablok regression show a nonparametric regression line for all markers (CD3, CD4, CD8, CD20, FOXP3 and CD68). The intercept is interpreted as the constant systematic bias (i.e. inaccuracy) component between the two observers. The slope measures the amount of proportional bias (i.e. inaccuracy) between the two observers.


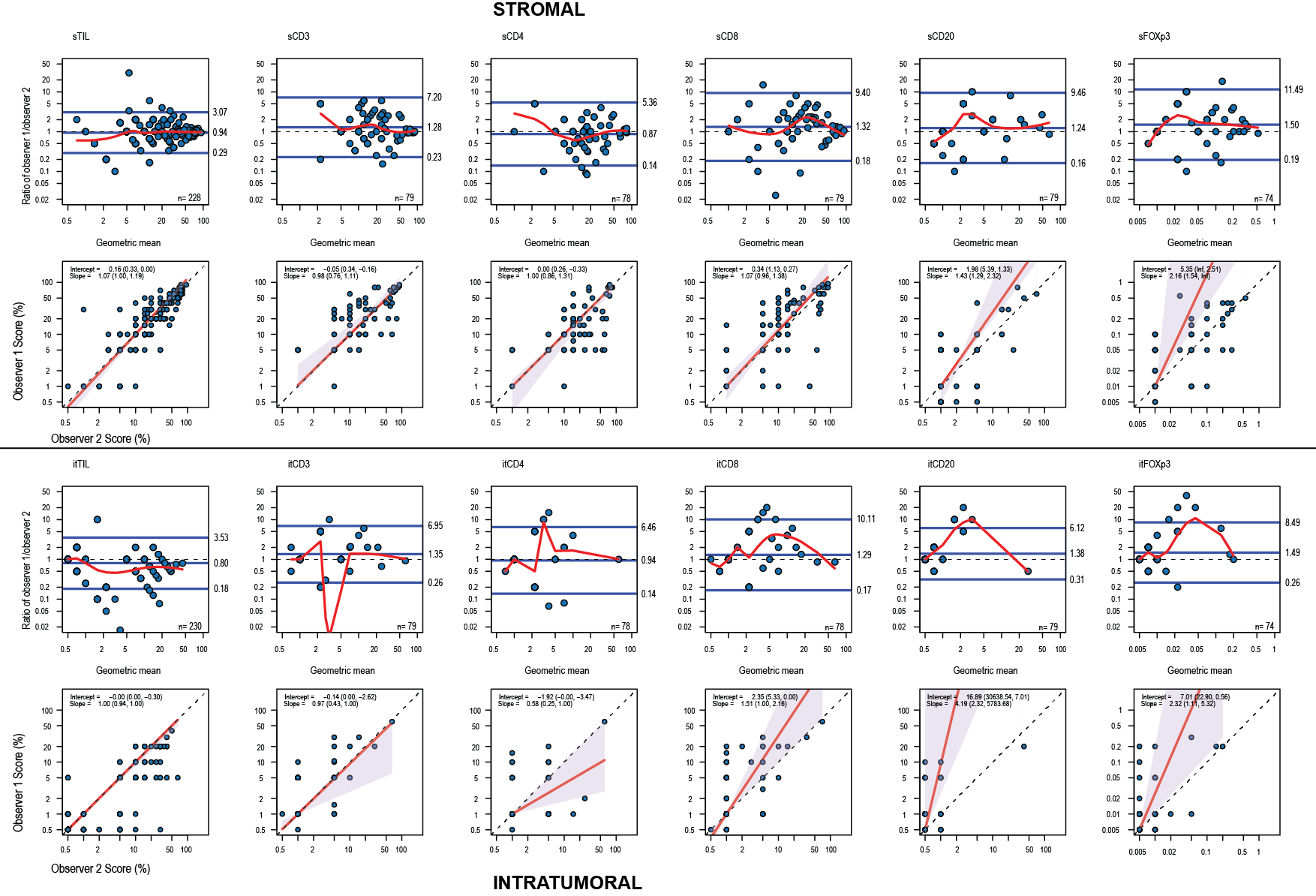


**Supplementary Figure 2.**

**Comparison between (dig)WS and (dig)TMA.**

**(a)** The Bland-Altman plots and Passing-Bablok regression analysis for comparing the amount of immune infiltrate scored on Whole Slides (WS) compared to Tissue Micro Array (TMA) for sTIL, sCD3, sCD4 and sCD8**.** **(b)** Forest plots for the CCC for TMA versus WS for all markers. **(c)** Bland-Altman analyses and Passing-Bablok regression for comparing digital CD3 scores on TMA and WS. **(d)** Bland-Altman analyses and Passing-Bablok regression for comparing CD3, CD4 and CD8 scoring by pathologists or the digital method on TMA.


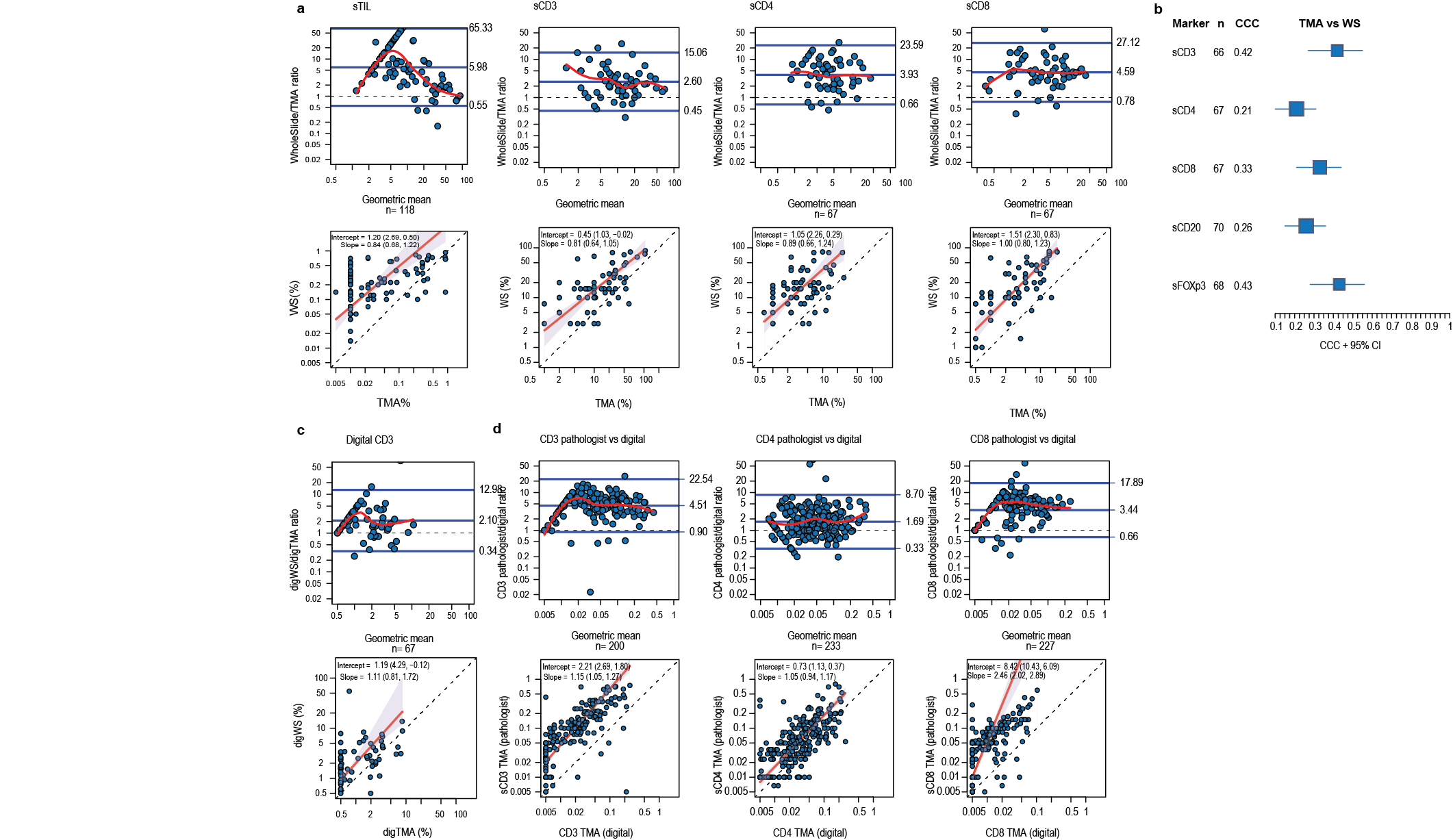


| Antibody | Clone | Species | Manufacturer | Platform | Dilution | HIER | Inc. time (min) | Detection |
| --- | --- | --- | --- | --- | --- | --- | --- | --- |
| Whole Slides | | | | | | | | |
| CD3 | SP7 | Rabbit | Thermo Scientific | Ventana Benchmark XT | 1/100 | CC1 standard | 60 | Universal DAB |
| CD4 | SP35 | Rabbit | Immunologic | Ventana Benchmark Ultra | RTU | CC1 24min | 32 | Optiview DAB |
| CD8 | C8/144B | Mouse | DAKO | Ventana Benchmark XT | 1/100 | CC1 standard | 60 | Universal DAB |
| CD20 | L26 | Mouse | DAKO | Ventana Benchmark XT | 1/750 | CC1 standard | 60 | Universal DAB |
| CD68 | PG-M1 | Mouse | DAKO | Ventana Benchmark XT | 1/200 | CC1 standard | 60 | Universal DAB |
| Foxp3 | 236A/E7 | Mouse | AbCam | Ventana Benchmark XT | 1/100 | CC1 standard | 60 | Universal DAB |
| Tissue Micro Array | | | | | | | | |
| CD3 | SP7 |  | Spring/ITK | Ventana Benchmark Ultra | 1/100 | CC1, 32 min | 32 | Optiview DAB |
| CD4 | SP35 |  | Cell Marque | Ventana Benchmark Ultra | 1/50 | CC1, 32 min | 32 | Optiview DAB |
| CD8 | C8/144B |  | DAKO/Agilent | Ventana Benchmark Ultra | 1/200 | CC1, 32 min | 32 | Optiview DAB |
| CD20 | L26 |  | DAKO/Agilent | Ventana Benchmark Ultra | 1/800 | CC1, 32 min | 32 | Optiview DAB |
| CD68 | KP1 |  | DAKO/Agilent | Ventana Benchmark Ultra | 1/10000 | CC1, 32 min | 32 | Optiview DAB |
| Foxp3 | 236A/E7 |  | AbCam | Ventana Benchmark Ultra | 1/200 | CC1, 64 min | 60 | Optiview DAB |

**D. Supplementary Tables**

**Supplementary Table 1.** The procedures, clones, manufacturer and dilution used for the immunohistochemistry on tissue micro array (TMA) and Whole Slides (WS).

| Method | Description |
| --- | --- |
| sTIL WS H&E | Stromal Tumor Infiltrating Lymphocytes, scored as the % of positive immune cells in the stromal compartment on a whole H&E slide. |
| iTIL WS H&E | Intratumoral Tumor Infiltrating Lymphocytes, scored as the % of positive immune cells in the intratumoral compartment on a whole H&E slide. |
| sTIL TMA H&E | Stromal Tumor Infiltrating Lymphocytes, scored as the % of positive immune cells in the stromal compartment of TMA H&E cores (average of multiple cores) |
| TIL TMA IHC | The % CD3+ and CD20+ positive immune cells in the TMA cores (average of multiple cores) |
| TIL WS IHC | The % CD3+ and CD20+ positive immune cells on the whole IHC slides |
| TIL digTMA IHC | The positive area of CD3+ and CD20+ positive immune cells calculated by Visiopharm compared to the tissue area in the TMA cores. A percentage is provided of the average of multiple cores. |
| metCBS (methylCIBERSORT)^17^ | A methylation-based deconvolution method that estimates the fraction of immune cell populations. |
| meTIL (methylation TIL)^11^ | A methylation-based signature for tumor infiltrating immune cells |
| EPIC^6^ | A gene expression-based deconvolution method to estimate the fraction of immune cell populations compared to the overall cellular fraction. |
| aCBS (absolute CIBERSORT)^4^ | A gene expression-based deconvolution method to estimate the fraction of immune cell populations, The ‘a’ before CBS indicates that the proportion was calculated with respect to the overall cellular fraction. |
| qSEQ (QuanTIseq)^5^ | A gene expression-based deconvolution method to estimate the fraction of immune cell populations compared to the overall cellular fraction. |
| xCELL ^14^ | Marker gene-based approach that utilizes a list of genes that are characteristic for each cell type. |
| MCP (MCP-counter)^18^ | Marker gene-based approach that utilizes a list of genes that are characteristic for each cell type. |
| CytAct^12^ | An immune signature for cytolytic activity. |
| IFNg^13^ | An immune signature for interferon-gamma signaling. |
| Davoli^16^ | Immune cell gene signature to express the relative abundance of a cell in a sample. |
| Danaher^15^ | Immune cell gene signature to express the relative abundance of a cell in a sample. |

**Supplementary Table 2.** Description of all methods used in the manuscript to estimate immune cell infiltration.

**Supplementary Table 3.** Overview of used cell fractions for overall immune infiltration and specific immune subtypes. If multiple cell types are listed for a method, these were summed.

| Method | Overall immune infiltration | T cells | CD4+ T cells | CD8+ T cells | Tregs | B cells | Macrophages |
| --- | --- | --- | --- | --- | --- | --- | --- |
| TMA IHC | CD3+CD20 | CD3 | CD4 | CD8 | Foxp3 | CD20 | CD68 |
| digTMA IHC | CD3+CD20 | CD3 | CD4 | CD8 | Foxp3 | CD20 | CD68 |
| WS IHC | CD3+CD20 | CD3 | CD4 | CD8 | Foxp3 | CD20 | CD68 |
| WS digital | NA | CD3 | NA | NA | NA | NA | NA |
| Absolute CIBERSORT  (aCBS) ^4^ | Summed T and B cells for absolute CIBERSORT version. Relative version: NA | T.cells.CD8, T.cells.CD4.naive, T.cells.CD4.memory.resting, T.cells.CD4.memory.activated, T.cells.follicular.helper,T.cells.regulatory, T.cells.gamma.delta | T.cells.CD4.naive,T.cells.CD4.memory.resting,  T.cells.CD4.memory.activated,T.cells.follicular.helper | T.cells.CD8 | T.cells.regulatory.Tregs. | B.cells.naive , B.cells.memory | Monocytes, Macrophages.M0, Macrophages.M1, Macrophages.M2 |
| quanTIseq, lsfit (qSEQ) ^5^ | CD4+ CD8+Treg+CD19 | T.cells.CD4 ,T.cells.CD8, Tregs | T.cells.CD4 | T.cells.CD8 | Tregs | B.cells | Macrophages.M1, Macrophages.M2, Monocytes |
| MCP-counter (MCP) ^18^ | NA | T.cells | Cytotoxic.lymphocytes | CD8.T.cells | NA | B.lineage | Monocytic.lineage |
| xCell ^14^ | NA | CD4.memory.T.cells,CD4.naive.T.cells, CD4.T.cells, CD4.Tcm, CD4.Tem, CD8.naive.T.cells, CD8.T.cells, CD8.Tcm,CD8.Tem, Tgd.cells, Th1.cells, Th2.cells, Tregs | CD4.memory.T.cells, CD4.naive.T.cells,CD4.T.cells,CD4.Tcm, CD4.Tem | CD8.T.cells,CD8.Tcm,CD8.Tem | Tregs | B.cells ,Memory.B.cells, naive.B.cells | Macrophages, Macrophages.M1, Macrophages.M2 |
| EPIC ^6^ | Bcells, CD4.Tcells,  CD8.Tcells | CD4.Tcells ,CD8.Tcells | CD4.Tcells | CD8.Tcells | NA | Bcells | Macrophages |
| MethylCIBERSORT (metCBS) ^17^ | CD8, CD19, CD4.Eff, Treg | CD4.Eff ,CD8 ,Treg | CD4.Eff | CD8 | Treg | CD19 | NA |
| Cell signatures Davoli et al. ^16^ | NA | NA | CD4.Tcells | CD8.Tcells | Tregs | B.cells | Macrophages |
| Cell signatures Danaher et al. ^15^ | NA | NA | Th1 | CD8.Tcells | Tregs | B.cells | Macrophages |
